# Impact of Visual and Auditory Relaxation Techniques on Autonomic Responses During Dental Procedures: A Randomized Controlled Trial

**DOI:** 10.7759/cureus.84422

**Published:** 2025-05-19

**Authors:** Yuriko Hayashida, Hanako Kawasaki, Takao Ayuse, Naomi Tanoue

**Affiliations:** 1 Department of Clinical Physiology, Graduate School of Biomedical Sciences, Nagasaki University, Nagasaki, JPN; 2 Developmental and Nurturing Dentistry, Graduate School of Biomedical Sciences, Nagasaki University, Nagasaki, JPN; 3 Clinical Research Center, Nagasaki University Hospital, Nagasaki, JPN

**Keywords:** autonomic nervous system, dental anxiety, dental phobia, psychotherapy, randomised controlled trial, relaxation therapy

## Abstract

Introduction: Relaxation methods for managing anxiety can help overcome and prevent dental treatment phobia. We investigated the effects of relaxation techniques targeting visual and auditory domains, which remain understudied, on dental treatment-related stress.

Methods: Thirty adult volunteers with dental anxiety were enrolled in a randomized, open-label, four-sequence, four-period crossover study evaluating the effects of the following interventions: wearing a hot eye mask with a heat- and steam-generating (HSG) sheet, wearing an inactivated HSG sheet, listening to nature sounds, and wearing noise-cancelling headphones. Interventions were separated by a washout period of at least two weeks to mitigate carry-over effects. Participants’ physiological parameters (autonomic nervous system function variables, skin conductance, and respiratory rate [RR] during dental treatment) were continuously monitored. The impact of these interventions on stress reduction was assessed.

Results: Application of a hot eye mask with an HSG sheet to the eye area reduced heart rate (HR) and increased the high-frequency component of HR variability (HRV) and skin conductance, indicating enhanced parasympathetic activity. Conversely, applying the inactivated HSG sheet tended to increase HR and low-frequency/high-frequency HRV ratios. Listening to nature sounds decreased RR and skin conductance and increased the high-frequency HRV component. Wearing noise-cancelling headphones increased the RR and decreased the high-frequency component of HRV.

Conclusions: Applying a hot eye mask with an HSG sheet and listening to nature sounds potentially reduced stress. However, applying an inactivated HSG sheet and using noise-cancelling headphones promoted sympathetic activity. Further research should subdivide anxiety scales and explore relaxation methods tailored to specific anxiety triggers.

## Introduction

Dental anxiety and phobia are closely related, but represent distinct pathologies [1]. In cases of dental anxiety, patients experience anticipatory anxiety before encountering a feared stimulus; however, they are still able to attend dental visits. Although treatment can proceed, it is often accompanied by elevated levels of tension and stress; this condition is sometimes referred to as mild dental phobia [2]. Dental phobia is characterized by avoidant behavior resulting from previous traumatic experiences or perceived danger. Intense fear can be triggered not only by actual exposure but also by the mere thought of the feared situation, and in severe cases, patients may exhibit significantly reduced oral health-related quality of life (OHRQOL) [3].

There is considerable individual variation in the perception of dental anxiety, and discrepancies often exist between patients’ self-reports and clinicians' assessments [4]. Various international criteria have been developed to assess dental anxiety, including the Index of Dental Anxiety and Fear (IDAF-4C+) [5] and Kleinknecht’s Dental Fear Survey (DFS) [6]. Although a Japanese version of the DFS exists [7], it is rarely used in clinical practice. In Japan, dental education offers limited instruction on dental anxiety and phobia, and the use of objective questionnaires to assess dental anxiety remains uncommon [8]. However, subjective assessment can result in the under-recognition or misdiagnosis of mild dental anxiety. In many cases, dental anxiety is recognized only after maladaptive behaviors emerge, such as refusing local anesthesia administration or unwillingness to open the mouth during treatment. By the time these behaviors are recognized, the condition may progress to moderate or severe dental phobia. In more severe cases, it may be accompanied by post-traumatic stress disorder (PTSD)-like symptoms, which make clinical management particularly challenging.

Various approaches are used to manage dental anxiety and phobia, including psychological interventions (e.g., relaxation techniques and cognitive-behavioral therapy) and pharmacological methods [2]. Sedatives and anxiolytics are often used in moderate to severe cases, with intravenous sedation providing high patient satisfaction owing to its anxiety-reducing effects. However, only a limited number of medical centers offer pharmacotherapy, which is not readily accessible upon request [9]. Additionally, we previously found that the "presence, absence, and frequency of intravenous sedation experiences in patients with dental anxiety were significantly associated with the incidence of dental caries during periods of treatment interruption" [10]. Patients with dental anxiety who experience anxiolytic, sedative, and amnestic effects of intravenous sedation may develop a psychological dependence on sedation and reduce awareness of caries risk. To overcome dental anxiety and phobia while preventing their progression, it is essential to implement early interventions that promote behavioral changes in patients.

Previous studies have shown that psychological interventions, while having less immediate effects than pharmacological interventions, have lower dropout rates when consistently used. Reductions in fear and anxiety can be maintained over time, enabling patients to manage their anxiety independently [2]. In addition, Aartman et al. found that in a follow-up study conducted one year after dental treatment, a smaller proportion of patients who received behavioral modification techniques exhibited higher levels of anxiety compared to those who underwent intravenous sedation. Managing anxiety using non-pharmacological behavioral management may also be superior to using anxiolytics [11]. Relaxation techniques are a form of psychological intervention. The methods commonly used for anxiety management include muscle relaxation, breathing exercises, and autogenic training. Although these methods are relatively simple, they require patient initiative and time to be effective [12].

Reducing sensory triggers in the dental environment and directing attention to alternative auditory or visual stimuli can also be effective in managing patient anxiety [9]. In the auditory domain, it has been suggested that music interventions and implementing noise cancellation can reduce dental anxiety [13-14]. However, most studies have focused on objective measures, such as visual analog scales, or on heart rate and blood pressure as indicators of autonomic nervous system activity. Few studies have combined multiple physiological measures to consider both the regulatory function of the autonomic nervous system and modulation of the stress response. In the visual domain, the use of virtual reality goggles is beneficial for distraction [2]. However, although the effects of blocking visual information [15-16] and localized thermotherapy have been demonstrated in the field of psychophysiology [17-19], few studies have tested their relaxation effects, specifically in terms of dental stress.

The purpose of this study was to quantify anxiety and stress levels and evaluate the effects of relaxation methods in the visual and auditory domain techniques proven to be effective in psychophysiology on dental-specific stress. We hypothesized that during dental treatment, any of the following interventions would activate the parasympathetic nervous system and effectively reduce stress: a hot eye mask with a heat- and steam-generating (HSG) sheet, blocking visual input, listening to nature sounds, or masking treatment noises.

## Materials and methods

The study complied with the Declaration of Helsinki and was reviewed and approved by the Research Ethics Committee of the Graduate School of Medical and Dental Sciences, Nagasaki University (application no. 22090101) on 27 October 2022. The study was registered in the UMIN Clinical Trials Registry (UMIN000049556) on November 18, 2022, and was conducted per the Consolidated Standards of Reporting Trials (CONSORT) 2010 guidelines and the CONSORT-Outcomes 2022 extension. Volunteers were recruited through notice boards at Nagasaki University Hospital and Nagasaki University. All participants were fully briefed on the aims and methods of the study and provided written informed consent before participation.

Study design

This was a randomized, open-label, four-sequence, four-period crossover study conducted at Nagasaki University Hospital between December 1, 2022, and November 30, 2023. We performed a preliminary power analysis using the G*Power 3.1 software package (Heinrich-Heine-Universität Düsseldorf, Düsseldorf, Germany). The sample size was determined based on Cohen's criterion [20] with an effect size of 0.25, a significance level of alpha of 0.05, and a power of 80%. Consequently, the required sample size was 24 participants.

Participants were randomized to one of four intervention groups using a mixed-effects model, based on the Williams design [21], to prevent selection bias: ADBC, BACD, CBDA, or DCAB (A, hot eye mask with an HSG sheet; B, inactivated HSG sheet; C, listening to nature sounds; and D, noise-cancelling headphones).

The four relaxation interventions used in this study were selected based on psychophysiological principles and clinical feasibility in dental settings. Warming the periocular area is expected to promote physical relaxation, as it has been associated with parasympathetic activation in psychiatric research. Covering the eye area with a towel or eye mask is hypothesized to reduce visual stimulation and induce psychological comfort. Listening to nature sounds was included because of their known stress-reducing properties. Noise-cancelling headphones were used to isolate environmental and dental treatment sounds and evaluate the physiological effects of auditory masking. All interventions were designed to target either the visual or auditory sensory pathways under the hypothesis that they may help modulate stress responses during dental procedures.

**Figure 1:**
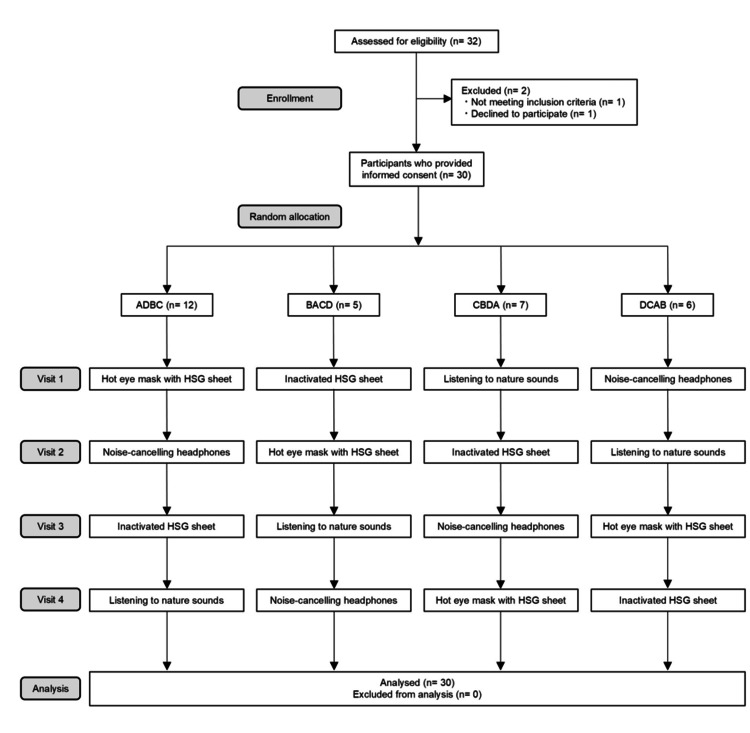
Consolidated Standards of Reporting Trials (CONSORT) diagram of the study. A flowchart of interventions is presented: ADBC, BACD, CBDA, or DCAB: A, hot eye mask with a heat- and steam-generating (HSG) sheet; B, inactivated HSG sheet; C, listening to nature sounds; D, noise-cancelling headphones. Each participant received all four types of relaxation intervention, with the order of administration randomly allocated.

Randomization was conducted using the RANDBETWEEN function in Microsoft® 365 Excel (Microsoft Corp., Redmond, WA), and participants were informed in advance of the order of interventions. Each intervention was separated by a washout period of at least 2 weeks to ensure that carryover relaxation effects did not occur between interventions. Figure 1 presents the CONSORT flowchart.

Participants

Before signing the consent form, participants were administered the Japanese version of the DFS, which objectively assesses dental anxiety and fear. The DFS is a screening test consisting of 20 items, each rated on a 5-point scale from *not at all* to *very much*. The total score ranges from 20 to 100, with higher scores indicating higher levels of anxiety and fear.

The inclusion criteria were healthy individuals aged 20-40 years, with a DFS score of 21 or higher. Participants were excluded if they had any of the following: conditions unsuitable for electrode gel 3 of 11 application due to skin diseases; those deemed unsuitable by researchers, those with mental illness or physical disabilities other than dental treatment phobia, those with sensitive teeth in the mandible, and those who withdrew during the study period. Ultimately, 30 participants who met the eligibility criteria were enrolled in this study.

Study protocol

Participants were instructed to avoid exercise before the measurements to prevent any impact on the physiological data. The temperature and humidity in the dental room were maintained at average levels of 25 °C and 45%, respectively. Participants were instructed to remain in a supine position on the dental unit with their eyes closed. Each participant wore the respective devices in the order determined by their group assignment. Biometric data were continuously measured throughout the study. Anxiety and stress elicit physiological responses such as psychogenic sweating, sympathetic activation, vagal withdrawal, and rapid shallow breathing. To quantitatively assess psychological sweating in response to stress-inducing stimuli, stress levels were measured by SC using a mobile eSense Skin Response (Mindfield Biosystems, Inc., Berlin, Germany) on an Apple iPhone. Electrodes were attached to the index and middle fingers of the left hand using Velcro straps, and data were exported to Excel for analysis. Respiratory rate (RR) was measured noninvasively and continuously using photoplethysmography with a Nellcor PM1000N (Medtronic, Inc., Minneapolis, MN) pulse oximeter sensor attached to the index finger of the right hand. Autonomic nervous system function was assessed using the MWM20 (GMS Inc., Tokyo, Japan). Real-time power spectrum analysis of heart rate (HR) variability (HRV) is used to evaluate autonomic balance, particularly the relationship between sympathetic and parasympathetic nervous activity in response to stress. In this study, HRV spectrum analysis was conducted using MWM20 analysis software on a Windows 11 computer via Bluetooth. Two EEG electrodes and one ECG electrode were attached to the forehead and chest, respectively. Alpha band power, high-frequency spectral power (HF; an index of parasympathetic nerve activity), and low-frequency spectral power (LF; influenced by both the sympathetic and parasympathetic nerves) of HRV, and HR were measured over time.

**Figure 2:**
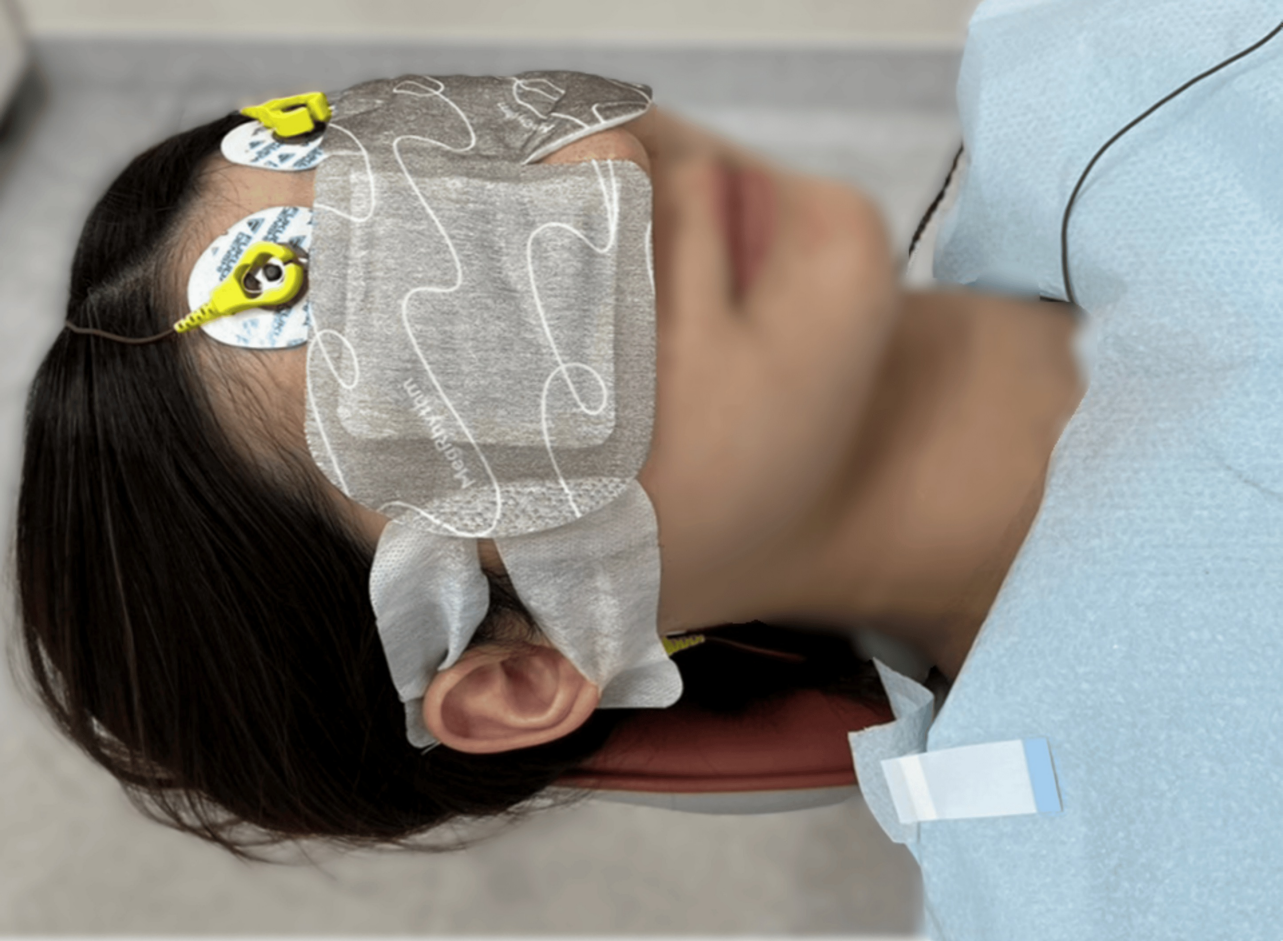
Diagram of interventions: hot eye mask with a heat- and steam-generating (HSG) sheet and inactivated HSG sheet.

Each experimental session consisted of the following four stages: (1) Participants wore either a hot eye mask with an HSG sheet (Kao Corp., Tokyo, Japan) (Figure 2), an inactivated HSG sheet (Figure 2), headphones through which they listened to nature sounds, or noise-cancelling headphones (MDR-1RNC; Sony Corp., Tokyo, Japan) and started the relaxation intervention. (2) Participants rested for 2 minutes. (3) Participants underwent a supragingival cleaning procedure on the mandibular teeth using a dental air scaler (Yoshida Dental Manufacturing Co., Ltd., Tokyo, Japan) for 2 minutes. (4) After the relaxation session, participants rested for 2 minutes. Individuals with dental anxiety or fear may experience stress simply by wearing a monitor in the dental treatment room. Therefore, to avoid distressing the study participants, no physiological measurements were taken during standard dental procedures in which no relaxation interventions were used.

Application of HSG Sheet

The hot eye mask with an HSG sheet used in this study was MegRhythm Steam thermal eye mask sheet fragrance-free (Kao Corp.). It consists of a non-woven fabric sheet containing iron powder as the heating element, generating heat through an oxidation reaction between the iron powder and oxygen. This heat caused water vapor to form from the water within the heating element, which reached the area around the eyes through the non-woven fabric. The sheet increased the skin temperature around the eyes to approximately 40 °C and could provide moist heat for approximately 20 minutes.

Application of Inactivated HSG Sheet

The inactivated HSG sheet used in this study was the hot eye masks with an HSG sheet that had been opened for 24 hours to allow complete heat dissipation. The size and material of the non-woven sheet in contact with the eyes were the same as those of the hot eye masks with the HSG sheets, making the two visually indistinguishable.

Application of Nature Sounds

The participants listened to nature sounds through headphones (MDR-1RNC, Sony Corp.), with the noise-cancelling function turned off. Nature sounds included those of the waves, wind, and birds. We downloaded and used an MP3 file of the *wild bird chorus* from the Sound Effect Lab website, which does not require attribution or a link for the use of audio material. To prevent discomfort, the volume was adjusted to the desired level (maximum of 65 dB) before starting measurements. Although headphones helped to block the sounds of dental treatment, they also hindered communication with the dentist. The start and end of dental treatment were signalled by tapping the oral cavity twice.

Application of Noise-Cancelling Headphones

In this study, headphones with digital noise cancellation (MDR-1RNC, Sony Corp.) were used. These were the same headphones as those used to listen to nature sounds. When the noise-cancelling function was activated, the built-in microphone detected and digitised the noise, which was cancelled out by the generation of an out-of-phase sound, significantly reducing the overall noise level.

Outcome and measurement

The primary outcome was the change in autonomic nervous system function (HF, LF/HF, alpha band power, and HR) during dental treatment across the four types of relaxation. The secondary outcome was the changes in SC and RR during dental treatment.

Statistical analysis

Statistical analyses were performed using the JMP Pro v.17 software package (SAS Institute Japan, Tokyo, Japan). Data distributions of quantitative variables during the 2-minute dental procedure were evaluated using repeated-measures analysis of variance (ANOVA) with mixed-effects models. To evaluate potential order-related effects on physiological responses, order, intervention timing, and relaxation condition were included as fixed effects in the mixed-effects model. Based on within-subject paired data, effects of the four relaxation interventions were assessed for differences in estimates and their associated 95% confidence intervals (CIs) using multiple comparison tests following repeated-measures ANOVA.

## Results

Thirty-two volunteers were screened for eligibility. Of these, one did not meet the inclusion criteria, and another declined to participate. Ultimately, 30 participants (17, 57%, women and 13, 43%, men) were enrolled in the study.

The demographic and descriptive characteristics of the participants are presented in Table 1. The participants were randomly assigned to one of four groups: ADBC (*n* = 12), BACD (*n* = 5), CBDA (*n* = 7), and DCAB (*n* = 6). No participants were excluded during the study; therefore, data from all participants were analyzed. The overall mean age was 27.7 years (standard deviation [SD]: 3.0), and the mean DFS score was 31.6 (SD 8.5).

**Table 1:** Description of the study sample (n = 30). Data are presented as frequencies (percentages) for categorical variables and as means (SD) and medians (interquartile range) for continuous variables. A: Wearing a hot eye mask with a heat- and steam-generating (HSG) sheet; B: Wearing an inactivated HSG sheet; C: Listening to nature sounds; D: Wearing noise-cancelling headphones. SD, standard deviation; DFS, Dental Fear Survey; DMFT index, Decayed, Missing, Filled Teeth index

Characteristics			Treatment sequence group (*n* = 30)									
			ADBC (*n* = 12)		BACD (*n* = 5)		CBDA (*n* = 7)		DCAB (*n* = 6)		Overall (*n* = 30)	
Sex												
	Male	*n* (%)	6	50%	2	40%	3	42.9%	2	33.3%	13	43.3%
	Female	*n* (%)	6	50%	3	60%	4	57.1%	4	66.7%	17	56.7%
Age (years)												
	Mean	SD	28.4	3.7	27.0	1.1	28.4	2.9	26.2	2.0	27.7	3.0
	Median	Range	28.0	23-37	27.0	25-28	28.0	25-34	25.5	24–30	27.0	23–37
DFS scores												
	Mean	SD	31.7	9.5	33.8	5.4	30.9	11.0	30.5	2.6	31.6	8.5
	Median	Range	28.5	21-50	32.0	27-43	25.0	22-55	31.5	25–33	30.5	21-55
DMFT index												
	Mean	SD	6.8	4.8	4.0	2.8	4.0	4.9	3.2	3.1	4.9	4.5
	Median	Range	7.5	0-16	4.0	0-7	1.0	0-14	2.0	0-9	4.5	0-16

Table 2 shows the effects of different interventions during dental procedures on various physiological parameters. HR decreased by an average of 2.70 beats per minute (95% CI: 0.39-5.02) under the hot eye mask with HSG sheet condition compared to the inactivated HSG sheet condition. Conversely, the inactivated HSG sheet condition tended to increase the HR compared to the other conditions. The most significant decrease in RR occurred under the listening to nature sounds condition. In contrast, wearing noise-cancelling headphones tended to increase the RR. Listening to nature sounds led to a change of 0.03 (95% CI: -0.24 to 0.31) in HF compared to wearing a hot eye mask with an HSG sheet, which was a marginal difference relative to other relaxation methods. HF levels tended to decrease when wearing noise-cancelling headphones compared to the other three relaxation interventions, although this difference was not statistically significant. LF/HF values tended to increase under the inactivated HSG sheet condition, while no significant difference in LF/HF was observed between the wearing noise-cancelling headphones and listening to nature sounds conditions. In terms of alpha band power, the listening to nature sounds condition produced a value of 0.14 (95% CI: -0.03 to 0.30) compared to the hot eye mask with an HSG sheet condition, 0.03 (95% CI: -0.14 to 0.19) compared to the inactivated HSG sheet condition, and -0.10 (95% CI: -0.26 to 0.07) compared to the wearing noise-cancelling headphones condition. For SC, wearing the hot eye mask with an HSG sheet tended to increase the values the most among the four relaxation methods. However, SC tended to decrease when listening to nature sounds compared to wearing the inactivated HSG sheet and noise-cancelling headphones. No adverse events were observed among participants across all interventions.

**Table 2:** Comparison of physiological parameters of the groups. The physiological parameters measured included heart rate (HR), respiratory rate (RR), high-frequency (HF) heart rate variability (HRV) component, low-frequency/high-frequency (LF/HF) HRV ratio, alpha band power, and skin conductance (SC). Data are expressed as estimated differences with 95% confidence intervals (CIs) and were analyzed using repeated-measures analysis of variance (ANOVA) followed by multiple comparisons. A summary of the expected direction of the effect of each intervention is provided below. Intervention A: ↓HR (relaxation), ↑SC (tension) Intervention B: ↑HR (tension), ↑LF/HF (relaxation) Intervention C: ↓RR (relaxation), ↓SC (relaxation) Intervention D: ↑RR (tension), ↓HF (tension) A: Wearing a hot eye mask with a heat- and steam-generating (HSG) sheet; B: Wearing an inactivated HSG sheet; C: Listening to nature sounds; D: Wearing noise-cancelling headphones.

Parameters	Interventions	Estimates difference	95% CI
Heart rate (beats/minute)	B-A	2.70	0.39-5.02
	C-A	0.80	-1.49 to 3.08
	C-B	-1.90	-4.22, 0.41
	D-A	1.25	-1.06, 3.56
	D-B	-1.45	-3.74, 0.84
	D-C	0.45	-1.86 to 2.77
Respiratory rate (beats/minute)	B-A	0.11	-0.97 to 1.20
	C-A	-0.53	-1.60 to 0.55
	C-B	-0.64	-1.73 to 0.45
	D-A	0.49	-0.60 to 1.57
	D-B	0.37	-0.70 to 1.45
	D-C	1.02	-0.07 to 2.10
High-frequency component	B-A	-0.15	-0.43 to 0.12
	C-A	0.03	-0.24 to 0.31
	C-B	0.19	-0.09 to 0.46
	D-A	-0.22	-0.50 to 0.05
	D-B	-0.07	-0.35 to 0.20
	D-C	-0.26	-0.54 to 0.02
LF/HF	B-A	0.10	-0.19 to 0.39
	C-A	-0.05	-0.34 to 0.24
	C-B	-0.15	-0.44 to 0.14
	D-A	-0.05	-0.34 to 0.24
	D-B	-0.15	-0.44 to 0.14
	D-C	0.00	-0.29 to 0.29
Alpha band power	B-A	0.11	-0.05 to 0.28
	C-A	0.14	-0.03 to 0.30
	C-B	0.03	-0.14 to 0.19
	D-A	0.04	-0.12 to 0.21
	D-B	-0.07	-0.23 to 0.10
	D-C	-0.10	-0.26 to 0.07
Skin conductance (μS)	B-A	-0.17	-0.48 to 0.13
	C-A	-0.24	-0.55 to 0.06
	C-B	-0.07	-0.38 to 0.24
	D-A	-0.16	-0.47 to 0.15
	D-B	0.01	-0.29 to 0.32
	D-C	0.09	-0.22 to 0.39

## Discussion

Our study results may be particularly helpful in designing early preventive interventions aimed at mitigating the escalation of dental fear in individuals with mild to moderate dental anxiety. The results of this study indicated that applying a hot eye mask with an HSG sheet to the eye area led to a decrease in HR and an increase in HF spectral power, which is an index of parasympathetic nervous activity, compared with the control condition, suggesting a strong relaxation effect in healthy individuals with mild to moderate dental anxiety. In particular, HR was reduced by 2.70 beats per minute with the hot eye mask as compared to that with the inactivated HSG sheet.

In a room temperature environment of approximately 25 °C, thermal stimulation of the skin at approximately 35-40 °C reportedly induces subjective comfort [22], whereas temperatures exceeding 43 °C are perceived as noxious stimuli [23]. The hot eye mask with an HSG sheet used in this study is a commonly available eyelid warming mask. With this device, the skin around the eye area is heated to approximately 40 °C, providing comfort through thermal sensory stimulation without causing pain. Previous research has demonstrated that wearing a hot eye mask with an HSG sheet stimulates the trigeminal nerve in healthy adults, suppresses sympathetic activity, and promotes parasympathetic activity, as evidenced by increased pupil change and miosis rate [17]. The changes in autonomic nerve activity observed in this study aligned with findings from other studies using HSG sheets on the abdomen, which also showed that this stimulus produced predominantly parasympathetic activity [18]. Another study that applied localized heat therapy to the eye area during mental arithmetic tasks in healthy adults reported that using the hot eye mask with an HSG sheet led to higher parasympathetic activity and reduced mental fatigue as compared to using an inactivated HSG sheet [19].

Anxiety is characterized by specific physiological responses such as sympathetic activation, vagus inactivation, and rapid, shallow breathing. Notably, increases in HR, LF spectral power, and the LF/HF ratio of HRV have been reported in individuals with anxiety [24]. Psychological sweating, which is linked to stress, fear, and tension, occurs in the hands and feet and is characterized by phasic secretions in response to stress stimuli [25]. Various electrodermal measurements have been employed to electrically capture psychological sweating, with the SC response (SCR) being one of the most commonly used measures. A higher SCR value indicates a greater level of emotional arousal. In this study, an increase in SC was observed when using a hot eye mask with an HSG sheet, which may be related to the stimulation of the autonomic nervous center and the subsequent local increase in skin temperature in remote areas such as the hands and feet [26]. Certain external environmental factors, such as temperature and humidity, can affect electrodermal measurement results [27]. Although room temperature was stable in this study, eye warming may have affected finger temperature and surface sweating, impacting the SC data.

Previous studies have shown that simply covering the eyes with an eye mask can reduce anxiety and tension scores [15]. In this study, when an inactivated HSG sheet was applied, the HR and the LF/HF ratio tended to increase. Short-term visual deprivation has been suggested to enhance auditory and tactile functions [16]. Although the inactivated HSG sheets used in this study were identical in size and material to the hot eye masks with an HSG sheet, the lack of thermal stimulation may have led to heightened tactile and auditory sensitivity during dental treatment, potentially resulting in increased anxiety and tension.

Listening to nature sounds decreased RR and SC and tended to increase HF, suggesting a stress-reducing effect, although this effect was not statistically significant. The difference in HF of HRV was negligible as compared to that in the hot eye mask with an HSG sheet condition. Music interventions have been reported to reduce anxiety in various medical fields. In dentistry, non-pharmacological methods may better manage more anxiety than anxiolytics [11]. Some studies have reported that music interventions can significantly reduce stress and lower HR, blood pressure, RR, pain sensation, and anxiety [13]. In the present study, similar to previous research, a tendency toward stress reduction was observed when listening to music during dental cleaning.

Using noise-cancelling headphones increased RR and decreased HF of HRV, suggesting parasympathetic suppression. The HRV spectrum analysis is a common psychophysiological index of stress-related emotional responses. HF reflects respiratory sinus arrhythmia and parasympathetic activity [28]. LF reflects Mayer wave fluctuations in systolic blood pressure and mediates both vagal and sympathetic activities. Because LF alone is inadequate for assessing sympathetic function, the LF/HF ratio is often used as a relative index of sympathetic activity. Participants were in a supine position with natural breathing, showing no arrhythmias or postural instability, suggesting minimal respiratory fluctuations. However, baseline measurements were not taken, and strict respiratory control was not implemented, limiting this study’s ability to exclude the influence of respiration on autonomic activity.

Vibrations applied to the teeth generate air- and bone-conducted noise, which are transmitted to the brain via the auditory nerve. Air conduction involves the collection of sound in the ear canal, which vibrates the eardrum and is converted into a nerve stimulus that is transmitted to the brain. Bone conduction vibrates the skull without involving the eardrum, causing relative motion between the footplate of the stapes and the oval window of the cochlea. This motion is transmitted directly to the cochlea in the inner ear, creating a nerve stimulus that is eventually perceived as a sound [29]. A previous study conducted on healthy adults in their 30s-50s found that wearing active noise-cancelling headphones could effectively reduce air-conducted noise during dental procedures and alleviate noise-related discomfort [14]. However, completely blocking bone-conducted noise using active noise-cancelling technology is challenging, suggesting that blocking external sounds may cause participants to focus on bone-conducted noise, leading to discomfort in some cases.

Regarding EEG measurements, no significant differences were observed between the various relaxation methods in this study. Generally, alpha band power is spontaneous potential fluctuation that commonly appears around the back of the head when an individual is at rest with eyes closed and relaxed. To measure EEG signals accurately, artifacts must be removed [30]. However, in this study, electromagnetic interference from other measuring devices, noise from electrical equipment used for cleaning, noise from commercial power sources, physiological artifacts, such as nystagmus and sweating, and static artifacts from clothing may not have been sufficiently removed, potentially influencing the results.

This study had some limitations. Although previous studies have shown that the four examined relaxation methods have stress-reducing effects, this study did not find any statistically significant differences. First, participants had mild to moderate dental anxiety, and measurements without relaxation interventions were omitted to prevent potential harm. Thus, it was not possible to determine the most effective method. Second, although a washout period of at least two weeks was implemented to minimize carryover effects, familiarity, learning, and fatigue between sessions may still have affected the results. Third, the largest difference in HR was observed between the hot eye mask with an HSG sheet and inactivated HSG sheet conditions; however, this study was unable to determine whether the tension associated with visual deprivation contributed to this effect. Fourth, many patients with dental anxiety and phobia were aware of specific factors that contributed to their anxiety and fear. Therefore, future research should compare the effects of using a hot eye mask with an HSG sheet and an inactivated HSG sheet in visually anxious individuals and compare the effects of listening to nature sounds and using noise-cancelling headphones in those with auditory anxiety in a randomized, open-label, two-group, two-period crossover study.

## Conclusions

The results of this study showed that applying a hot eye mask with an HSG sheet during dental treatment led to a decrease in HR, increase in the HF component of HRV, and increase in SC. When an inactivated HSG sheet was used, the HR and LF/HF ratio of HRV tended to increase. Listening to nature sounds resulted in decreases in RR and SC, with tendencies for HF and alpha band power to increase. Conversely, the use of noise-cancelling headphones was associated with an increase in the RR and a decrease in the HF of HRV. These findings suggested that the application of a hot eye mask with an HSG sheet and listening to nature sounds may have stress-reducing effects during dental treatments, while the use of an inactivated HSG sheet and noise-cancelling headphones may suppress parasympathetic nervous activity and favor sympathetic nervous activity during such treatment. While our findings suggest that certain relaxation techniques reduce stress, limitations such as the small sample size and lack of a control group necessitate caution in interpreting the results. While our findings suggest that certain relaxation techniques may help reduce stress, this study has limitations, including a small sample size and the absence of a control group. The observed effects were specific to individuals with mild to moderate dental anxiety and may not be generalizable to those with high levels of dental anxiety or phobia. Therefore, caution should be exercised when applying these findings to a larger population. Nonetheless, the results may offer valuable insights for designing early preventive interventions aimed at preventing escalation of dental fear.
